# Evaluating the effect of vitamin D supplementation on serum levels of 25-hydroxy vitamin D, 1,25-dihydroxy vitamin D, parathyroid hormone and renin–angiotensin–aldosterone system: a systematic review and meta-analysis of clinical trials

**DOI:** 10.1186/s40795-023-00786-x

**Published:** 2023-11-15

**Authors:** Alireza Khodadadiyan, Mahdi Rahmanian, Dorsa Shekouh, Melika Golmohammadi, Arshin Ghaedi, Aida Bazrgar, Mehrab Sayadi, Mehdi Bazrafshan, Aigin Heydari, Hamed Bazrafshan Drissi

**Affiliations:** 1 Student Research Committee, School of Medicine, Shiraz University of Medical Sciences, Shiraz, Iran; 2 Student Research Committee, Shahid Beheshti University of Medical Sciences, Tehran, Iran; 3 Cardiovascular Research Center, Shiraz University of Medical Science, Shiraz, Iran

**Keywords:** Ergocalciferols, Calcitriol, Vitamin D, Renin-Angiotensin System

## Abstract

**Background:**

Vitamin D, one of the most essential micronutrients, is crucial in various health outcomes. However, previous studies showed conflicting results and uncertainty about vitamin D supplementation's optimal dosage and duration. In this study, we aimed to evaluate the vitamin D supplements efficiency on serum levels of 25-hydroxy vitamin D (25(OH)D), 1,25-dihdroxy vitamin D (1,25(OH)2D), parathyroid hormone (PTH) and renin–angiotensin–aldosterone system (RAAS) in adults.

**Methods:**

A systematic analysis of eligible and relevant randomized-controlled trials (RCT) published before April 2023 assessing the effect of vitamin D supplementations applied. The studies were identified by searching several databases, including Pubmed, Scopus, Web of Science, ProQuest, and Cochrane Register of controlled trials.

**Results:**

Five eligible RCTs with 346 participants in the intervention and 352 participants in the control group were assessed in our project. According to the results, there was a substantial change in 25(OH)D (SMD: 2.2, *I*^*2*^: 92.3, 95% Confidence Interval (CI): 1.38–3.02, P-value: 0.048) and 1,25(OH)2D (SMD:1.23, *I*^*2*^: 86.3, 95% CI: 0.01- 2.44, *P*-value < 0.010) affected by vitamin D intervention. Regarding Parathyroid hormone (PTH), however, vitamin D intervention showed a remarkable decrease (SMD: -0.75, *I*^*2*^: 82.4, 95% CI: (-1.3)—(-0.18), *P*-value < 0.010). Moreover, sensitivity analysis showed significant publication bias in terms of 25(OH)D.

**Conclusion:**

Vitamin D supplements significantly increase the serum levels of 25(OH)D and 1,25(OH)2D and decrease PTH levels. While some studies reported decreasing effect of vitamin D supplements on RAAS activity, some reported no changes.

## Introduction

 Vitamin D deficiency is a prevalent major health issue worldwide, affecting up to 41.6% of adults in the US, and its prevalence is higher in non-Hispanic blacks (82.1%) and Hispanics (69.2%) [1]. Regarding several studies conducted in Iran, the prevalence of vitamin D deficiency among the adult population revealed that in different regions, the prevalence is between 50 and almost 95 percent [2, 3].

Vitamin D is essential to maintaining bone health, immune function, and overall well-being. Deficiency of this vitamin has been associated with several health conditions, such as osteoporosis, cardiovascular diseases, and breast cancer [4–6]. For instance, patients with breast cancer experienced adverse effects on their oral health due to vitamin D deficiency [7]. Inadequate serum levels of 25-hydroxyvitamin D (25(OH)D) have been intricately associated substantially with decreased strength in appendicular muscles and impaired physical performance, and it has been indicated to be linked to an augmented vulnerability to immune-related disorders, including psoriasis and autoimmune diseases. [8–10].

Vitamin D, a fat-soluble vitamin, has been suggested to play a potential role in preventing hypertension, stroke, heart failure (HF), and other metabolic and cardiovascular diseases (CVD), as the prevalence of its insufficiency is widespread among patients diagnosed with CVD, especially HF, and has been linked to the occurrence of cardiovascular events and heightened mortality rates [11, 12]. Several studies, encompassing both animal and human trials, propose that a lack of vitamin D results in an increased production of renin and the activation of the Renin-Angiotensin System (RAS), leading to potential damage to the kidneys and cardiovascular system. A hypothesized mechanism connecting vitamin D to hypertension suggests that vitamin D suppresses the RAS, influencing blood pressure regulation [13–15]. Therefore, vitamin D supplementation has become a popular strategy for maintaining adequate vitamin D levels in the body.

A number of randomized controlled trials (RCTs) tried to explore the impact of vitamin D supplementation on different health outcomes [16]. However, the results have been conflicting, and the optimal dosage and duration of vitamin D supplementation still need to be clarified [17]. Therefore, this systematic review and meta-analysis aims to assess the effectiveness of vitamin D supplementation on serum levels of 25(OH)D, 1,25-dihydroxy vitamin D (1,25(OH)2D), parathyroid hormone (PTH), and the Renin–Angiotensin–Aldosterone system in adults.

## Methods

This systematic review was conducted following the Preferred Reporting Items for Systematic Review and Meta-analysis (PRISMA) statement and was registered at PROSPERO (International prospective register of systematic reviews, ID = CRD42022360567) [18]. OpenAI ChatGPT was used for data extraction and clarification in the primary screening of the articles.

### Databases and search strategy

The computerized PubMed, Scopus, Web of Science, ProQuest, and Cochrane Register of controlled trials databases were systematically searched for relevant studies. The search was conducted between 27^th^ April and 29^th^ April 2023, using the strategy provided in the Supplementary File. The search strategy was modified appropriately for each database.

### Population

Patients over the age of 18 years, suffering from cardiovascular disease, were of interest. No limitations were considered regarding sex, nationality, the type of cardiovascular disease, the time from onset, or the medication in use.

### Intervention

Our focus was on the studies in which the vitamin D supplementation was the main intervention.

### Comparator

The studies using placebo, or continuing the standard treatment as a comparison group was considered. The presence of the control group was not limited.

### Outcome

As the primary outcome, we include those studies discussing about RAS activity, including plasma renin concentration (PRC), and parathyroid hormone (PTH) as measures. Furthermore, the secondary outcome of interest was occurrence of adverse events.

### Study selection

After the automatic removal of the duplicate studies using the tools provided by the 20^th^ version of Endnote, reviews, editorials, letters to the editor, theses, abstracts, case series, and case reports were excluded; peer-reviewed studies with interventional design, including randomized controlled trials (RCTs) and quasi-experimental studies evaluating the effect of vitamin D supplementation on the renin-angiotensin system, were included. Two reviewers (AKh and DSh) independently screened the title and abstract for relevant studies, and then another reviewer (AGh) screened the full text of the studies, considering the eligibility criteria. Both screening rounds were conducted under the fourth reviewer's (MG) supervision.

### Risk of bias assessment

Two reviewers independently assessed the risk of bias in the studies (AKh and DSh). To assess the selection bias, performance bias, detection bias, attrition bias, reporting bias, and other possible biases, the Cochrane Collaboration's assessment tool was used [19]. The third reviewer (MG) acted as a supervisor if a consensus could not be reached.

### Data extraction

The data extraction form included the study’s first author, publish year, country, study design, the total number of participants and divided numbers as intervention and control arms, the total and divided age of intervention and control group (mean ± standard deviation), the sex of the participants, the type of vitamin D supplementary and the dosage use, the therapy duration and follow up, probable second intervention, side effects, the primary outcome and measure, and ultimately, the mean and standard deviation of 25(OH)D and 1,25(OH)_2_D as vitamin D measures, and the mean and standard deviation of renin and aldosterone before and after intervention as renin-angiotensin system measures.

### Data analysis

Review Manager (RevMan) version 5.3 (Nordic Cochrane Centre, The Cochrane Collaboration, Copenhagen, Denmark) was utilized to analyze the data. Meta-analysis was run via STATA 13 (College Station, TX, USA). Standardized mean difference (SMD) was considered the effect size for comparing the mean change of variables between the placebo and intervention groups. The I-squared statistic indicated the amount of heterogeneity in the studies. The random effect model was used, where heterogeneity was significant at the 0.05 level. Also, the forest plot was provided for each study, and Egger's test assessed pooled data publication bias. The P-value extracted from this test was compared with a 0.05 significance level. Also, the 95% confidence interval was provided for the bias value. In addition, sensitivity analysis was done.

## Results

**Fig. 1:**
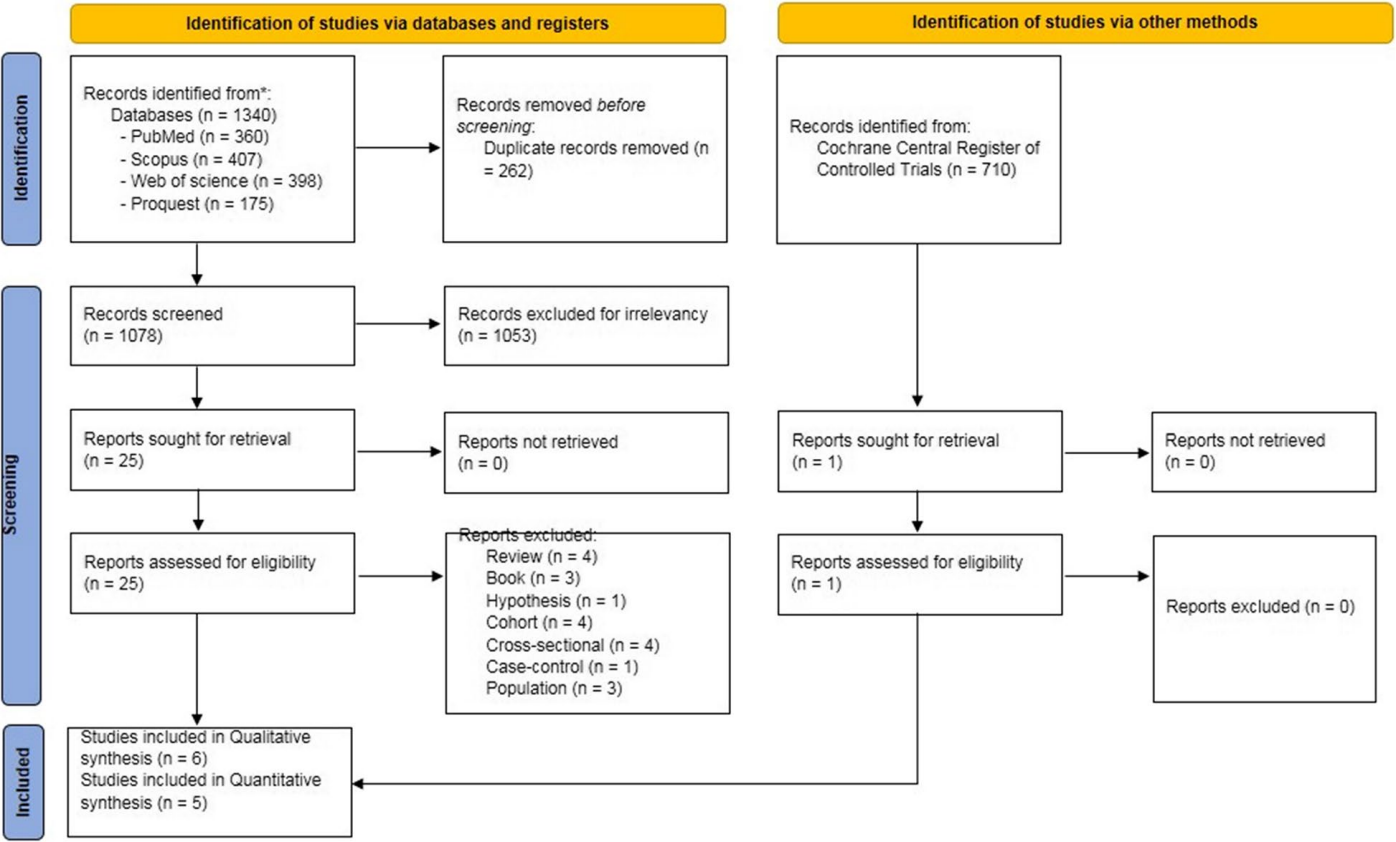
Study selection process flow diagram. *Consider, if feasible to do so, reporting the number of records identified from each database or register searched (rather than the total number across all databases/registers). **If automation tools were used, indicate how many records were excluded by a human and how many were excluded by automation tools

A total of 1078 published papers were collected after the primary screening, and ultimately, 5 RCTs [20–23, 25] with a total of 346 people in the intervention group and 352 in the control group (698 patients as a whole) were included after a precise review of the title and abstract followed by full text. After eliminating 262 duplicate documents, non-RCTs, and studies that failed to meet the inclusion criteria, six studies [20–25] were included in the qualitative analysis. Likewise, one of these studies [24] could not be analyzed quantitatively. Figure 1 clarifies the screening process.

**Fig. 2:**
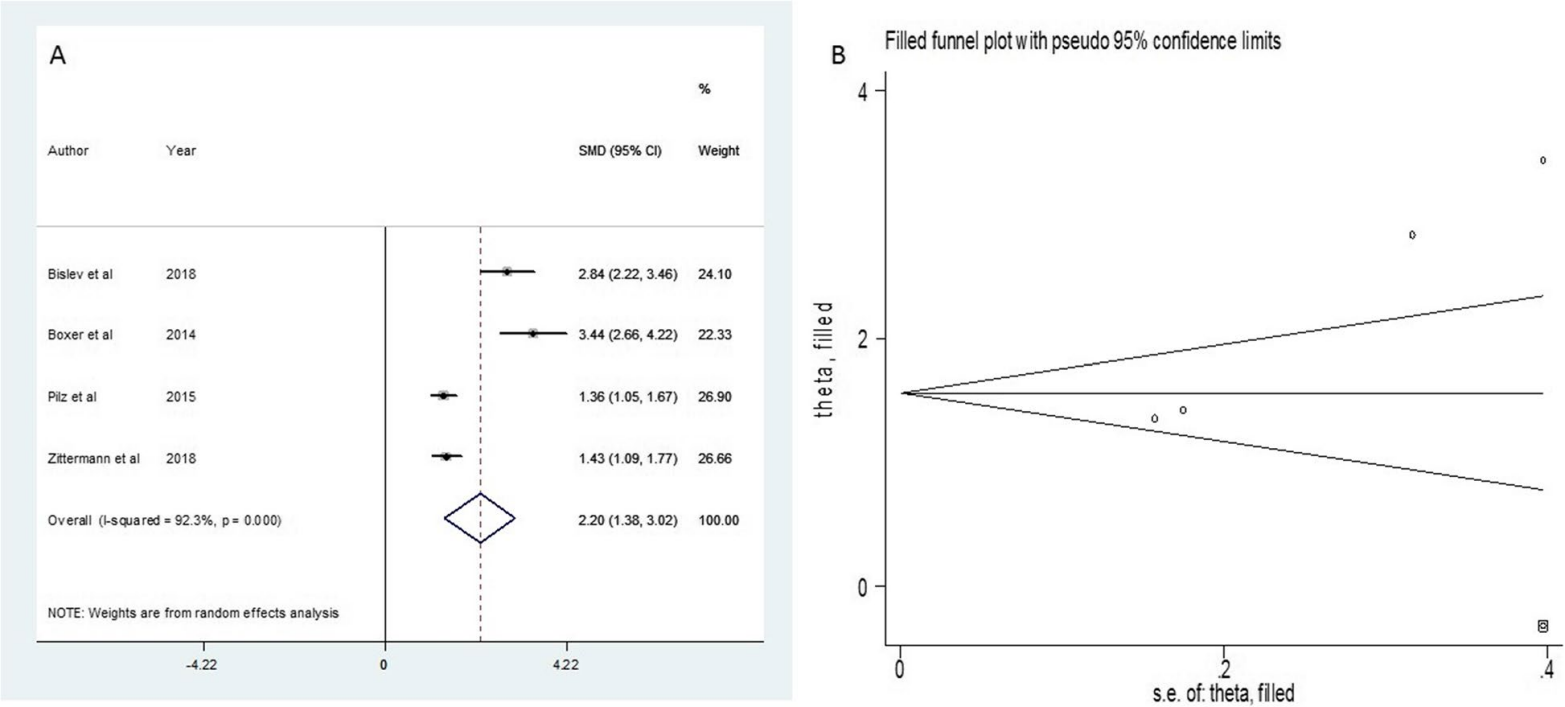
Funnel plot and standard mean difference (SMD) of change of 25(OH)D in two groups

All six of the selected studies [20–25] demonstrated an appropriate randomization approach; four of them adopted allocation concealment [20, 22, 23, 25], All of them [20–23, 25] utilized a double-blind method, and consequently, three [17, 18, 20] of them were free of any other biases (Figs. 1 and 2).

### Critical appraisal (Quality Assessment)

A glance at the quality assessment (Fig. 2) reveals striking differences between Bislev et al.’s study at the highest quality and Schroten et al.’s study at the lowest quality [20, 24]. Furthermore, since Schroten et al. had not utilized double-blind method alongside with having notable limitations including too short follow-up in a small trial, we eliminated that for meta-analysis.

### Included studies and population’s characteristics

 The mean age of the patients in the intervention and control groups is mentioned in Table 1. According to the results mentioned above (Table 1), the t-test revealed no significant statistical difference in patient age between the two groups. Sexuality was also the same between the two groups. The study details and the basic information about the population are indicated in Tables 1 and 2.

**Table 1:** Study details

ID	Author	Publication year	Country	Design	Blinding	Sample size	Exposure	Length of follow-up	Study Population	Significant results
1	Bislev, L. S. et al	2018	Aarhus, Denmark	Prospective RCT	Double-blind	81	vitamin D3 70 µg/day (2800 IU/day)	12 weeks	postmenopausal women aged 60–80 with secondary hyperparathyroidism (PTH > 6.9 pmol/l) and 25-hydroxy-vitamin D (25(OH)D) levels < 50 nmol/l	No changes in plasma aldosterone concentration (PAC) and plasma renin concentration (PRC), Increased high density lipoprotein (HDL), no effects on RAAS activity, reduced PTH
2	Boxer, R. S. et al	2014	United states of America	Prospective RCT	Double-blind	64	50,000 IU vitamin D3 per week	6 months	Mean age [SD] = 65.9 ± 10.4 years, NYHA class II–IV, 25(OH)D < 37.5 ng/mL	Decreased PAC ends up down regulating RAAS activity
3	Grubler, M. R. et al	2016	Austria	Prospective RCT	Double-blind	188	2800 IU of vitamin D3 as seven oily drops per day	8 weeks	Mean age [SD] = 60.1 ± 11.3 years with arterial hypertension and a 25(OH)D serum concentration below 30 ng/mL	Meaningful decrease in PAC, no effect on PRC
4	Pliz, S. et al	2015	Austria	Prospective RCT	Double-blind	188	2800 IU vitamin D3 as 7 oily drops per day	8 weeks	Mean age [SD] = 60.1 ± 11.3 years with arterial hypertension and a 25(OH)D serum concentration below 30 ng/mL	Significant increase in triglycerides but no significant effect on BP and several cardiovascular risk factors
5	Schroten, N. F. et al	2013	Netherlands	Prospective RCT	Single-blind	101	2000 IU oral daily	6 weeks	Mean age [SD] = 64 ± 10 years with CHF (left ventricular ejection fraction [LVEF] b45%)	Decrease in plasma renin activity (PRA) and PRC
6	Zitterman, A. et al	2018	North Rhine-Westphalia, Germany	Prospective RCT	Double-blind	165	4000 IU daily	8 weeks	Vit D group mean age [SD] = 54.5 ± 9.7 years, placebo mean age [SD] = 51.3 ± 10 years advanced heart failure	Increase in PRC and PAC, no effect on RAAS activity

Total number of cases were 346 (50.2% female) with mean age of 59.78 ± 10.6 and total number of controls were 352 (51.7% female) with mean age of 60.27 ± 10.8. There was no statistical difference between cases and controls regarding age (*p* = 0.518) and gender (0.674)

**Table 2:** Basic information of the population

Articles	**Intervention**	**N**	**Calcium (mmol/L)**				**25(OH)D nmol/L**				**1,25(OH)2D (pmol/L)**			
			Amount	Mean changes	*P*-value(changes)	*P*-value	Amount	Mean changes	*P*-value(changes)	*P*-value	Amount	Mean changes	*P*-value(changes)	*P*-value
Bislev 2018 [20]	Placebo	41	3.5 (2.7 to 5.2)	0.0 (− 8.6 to 8.6)	< 0.0001	NA	33 ± 9	− 13 (− 18 to − 8)	< 0.00001	NA	53 ± 13	− 17 (− 22 to − 11)	< 0.00001	NA
	Vitamin D	40	3.5 (2.7 to 5.2)	32.3 (18.0 to 46.7)			33 ± 9	199 (167 to 234)		NA	53 ± 13	42 (29 to 55)		NA
Boxer 2014 [21]	Placebo	33	9.2 ± 0.4	NA	NA	NA	17.8 ± 9	− 0.2 ± 6.6	0.001	NA	NA	NA	NA	NA
	Vitamin D	31	9.3 ± 0.5	NA	NA	NA	19.1 ± 9.3	42.3 ± 16.4		NA	NA	NA		NA
Grubler 2016 [22]	Placebo	95	2.37 ± 0.11	NA	NA	NA	20.4 ± 5.7 ng/mL		< 0.001	NA	NA	NA	NA	NA
	Vitamin D	92	2.37 ± 0.1	NA	NA	NA	21.8 ± 5.4 ng/mL			NA	NA	NA		NA
Pilz 2015 [23]	Placebo	100	2.37 ± 0.11	− 0.01 (− 0.03 to 0.01)	0.259	NA	20.4 ± 5.7	3.3 (1.8 to 4.7)	< 0.001	0.86	NA	NA	NA	NA
	Vitamin D	100	2.37 ± 0.10	0.00 (− 0.02 to 0.01)		NA	22 ± 5.5	14.2 (12.5 to 15.8)		NA	NA	NA		NA
Schroten 2013 [24]	Placebo	51	2.3 ± 0.1	− 0.01	0.1	0.85	46 (39–63)	44 (39–49)	< 0.001	NA	142(117–170)	132 (121–143)	< 0.001	0.8
	Vitamin D	50	2.3 ± 0.1	0.02			48(38–61)	80 (75–87)		NA	133(107–168)	194 (179–211)		NA
Zittermann 2018 [25]	Placebo	82	2.38 ± 0.11	NA	NA	0.634	36.7 (32.8–40.6)	9.6 (3.8 to 15.4)	< 0.001	0.667	90.1 ± 33.5	− 8.1 (− 15.8 to − 0.6)	< 0.001	0.252
	Vitamin D	83	2.38 ± 0.11	NA	NA	NA	36.9 (32.8–40.9)	65.8 (54.5 to 77.1)		NA	83.9 ± 34.3	12.2 (3.2 to 21.3)		NA
Articles	Intervention	N	PAC (ng/dl)											
			Amount				Mean changes				P-value(changes)		P-value	
Bislev 2018 [20]	Placebo	41	38 (27 to 56)				2.6 (− 14 to 22.3)				NA		0.93	
	Vitamin D	40	39 (27 to 56)				3.6 (− 10.6 to 20.1)							
Boxer 2014 [21]	Placebo	33	NA				NA				NA		NA	
	Vitamin D	31	NA				NA				NA		NA	
Grubler 2016 [22]	Placebo	95	16.8 ± 10.5				3.24				NA		0.04	
	Vitamin D	92	16.8 ± 10.6				0.88				NA			
Pilz 2015 [23]	Placebo	100	14.5 (10.5–19.7				3.3 (1.5 to 5.0)				NA		0.125	
	Vitamin D	100	15.1 (9.5–19.1)				0.9 (− 1.0 to 2.8)							
Schroten 2013 [24]	Placebo	51	NA				NA				NA		NA	
	Vitamin D	50	NA				NA							
Zittermann 2018 [25]	Placebo	82	NA				NA				NA		NA	
	Vitamin D	83	NA				NA				NA		NA	

Articles	**PRC (mIU/L) (ng/L)(µUmL) (pg/ml)**				**PRA (ng/mL per h)**				
	Amount	Mean changes	*P*-value(changes)	*P*-value	Amount	Mean changes	*P*-value(changes)	*P*-value	*P*-value
Bislev 2018 [20]	4 (2.6 to 6.4) (pg/ml)	− 1.8 (23.2 to 25.7)(pg/ml)	0.55	NA	NA	NA	NA	NA	NA
	4 (2.6 to 6.4)	7.1 (− 7.6 to 24.2)(pg/ml)		NA	NA	NA	NA	NA	NA
Boxer 2014 [21]	NA	NA	NA	NA	6.7 ± 8.6	9.2 ± 8	0.2	NA	NA
	NA	NA	NA	NA	7.6 ± 13.4	6.3 ± 7.0	NA	NA	NA
Grubler 2016 [22]	16.1 (9.5–51.6) µUmL	28.79 µUmL	NA	0.41	NA	NA	NA	NA	NA
	16.3 (10.2–38.7) µUmL	-1.54 µUmL	NA	NA	NA	NA	NA	NA	NA
Pilz 2015 [23]	16.7 (9.3–53.7) (µU/mL)	28.8 (− 23.0 to 80.6)(µU/mL)	0.128	NA	NA	NA	NA	NA	NA
	15.5 (9.6–35.8)(µU/mL)	− 1.5 (− 8.1 to 5.0)(µU/mL)		NA	NA	NA	NA	NA	NA
Schroten 2013 [24]	67(17–181) (ng/L)	72 (47–111)(ng/L)	0.02	0.76	4.5 (1.4–17.5)	7.3 (4.5–11.8)	0.002	0.46	0.45
	57(21–193)(ng/L)	55 (32–93)(ng/L)			5.4(2.5–28.1)	5.2 (2.9–9.5)			
Zittermann 2018 [25]	297 (141–752) (mIU/L)	NA	NA	0.954	NA	NA	NA	NA	NA
	300 (79–1277) (mIU/L)	NA	NA	NA	NA	NA	NA	NA	NA
Articles	PTH (pmol/l) (pg/mL)								
	Amount		Mean changes		P-value(changes)		P-value		
Bislev 2018 [20]	6.1 (5.1 to 6.9)		5.5 (1.3 to 9.8)		< 0.00001		NA		
	6.1 (5.1 to 6.9)		− 11.4 (− 15.6 to − 7.2)				NA		
Boxer 2014 [21]	72.8 ± 40.2(pg/mL)		− 3.1 ± 38.1		0.01		NA		
	62.3 ± 44.3		− 23.1 ± 40.0				NA		
Grubler 2016 [22]	NA		NA		NA		NA		
	NA		NA		NA		NA		
Pilz 2015 [23]	51.3 (38.8–63.7)		1.7 (− 1.2 to 4.7)		0.003		NA		
	49.0 (40.0–61.5)		− 4.0 (− 6.5 to − 1.6)				NA		
Schroten 2013 [24]	7.0 (4.4–9.2)		0.3(0.1–0.4)		0.004		0.45		
	7.8 (4.7–10)		− 1.7(− 2.1 to − 0.3						
Zittermann 2018 [25]	NA		NA		NA		NA		
	NA		NA		NA		NA		

*PRC* Plasma-renin concentration, *PRA* Plasma-renin activity, *PAC* Plasma-aldosterone concentration, *PTH* Parathyroid hormone, *NA* Not available

### Vitamin D and serum levels of 25(OH)D, and 1,25(OH)_2_D

**Fig. 3:**
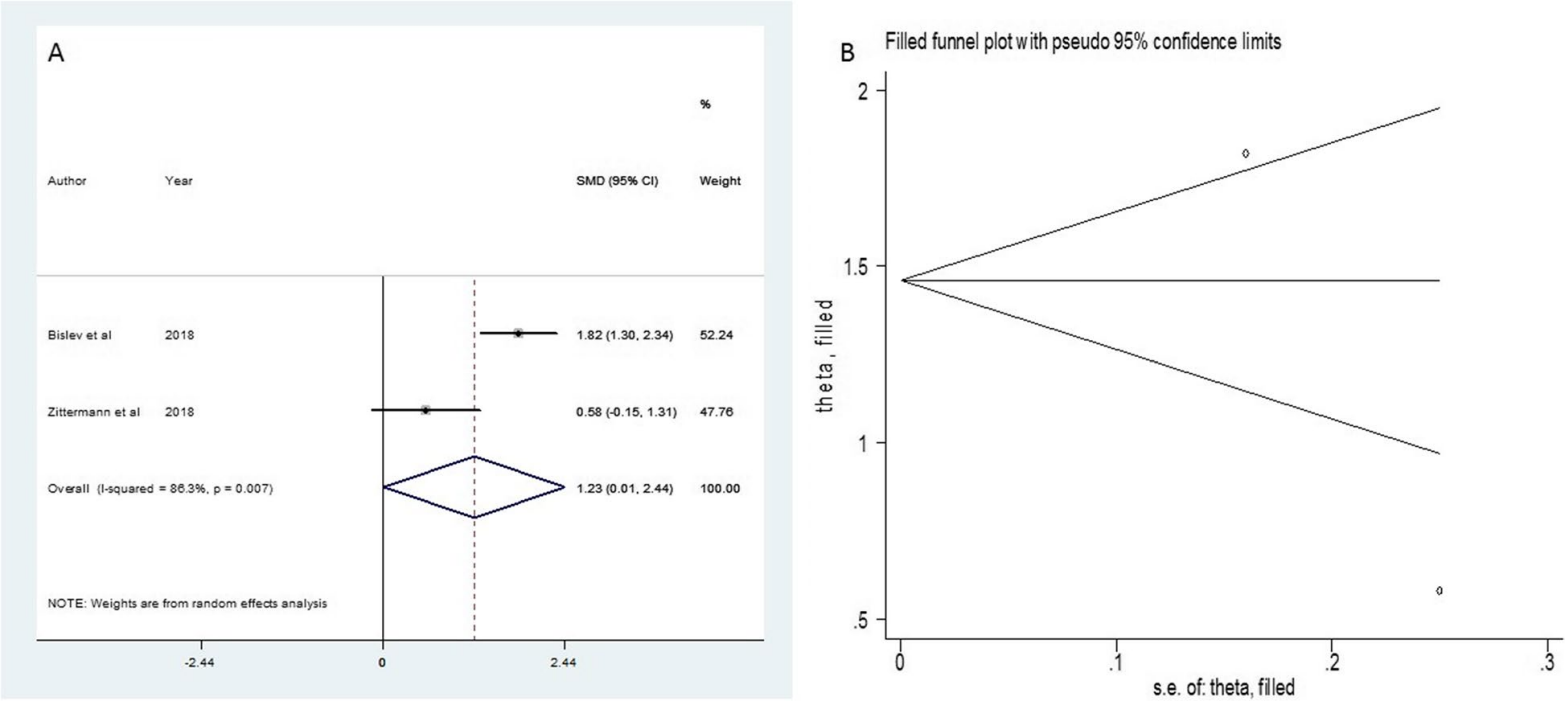
Funnel plot and standard mean difference (SMD) of change of 1.25(OH)D in two groups

**Fig. 4:**
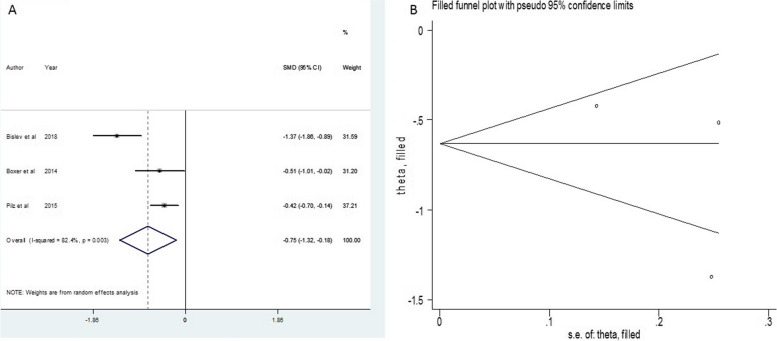
Standard mean difference (SMD) of change of PTH in two groups

The results of the standardized mean difference (SMD) for the changes in the parameters studied are included in Table 3. Given the statistically significant heterogeneity observed in the variables 25(OH)D and 1,25(OH)_2_D, the random effect model was used. That was in favor of the intervention group. The forest plot figure and funnel plots for assessing publication bias for all three parameters were calculated according to the studies involved, the results of which are elucidated in Figs. 2, 3, and 4. These figures identify studies subject to SMD size calculation. As illustrated in Table 1, vitamin D intervention led to a robust change in 25(OH)D and 1,25(OH)_2_D (SMD = 2.2, *I*^*2*^ = 92.3, 95% Confidence interval = 1.38–3.02, *P* value =  < 0.001 and SMD = 1.23, *I*^*2*^ = 86.3, 95% Confidence interval = 0.01–2.44, P value = 0.048, respectively).

**Table 3:** Combined SMD between intervention and control grope according to change of before and post as well as before and after intervention with heterogeneity and egger’s test

Variables	Model	Combined				Heterogeneity			Egger’s test		
		SMD	95%CI	Z for SMD = 0	*P* value	*I*^*2*^*(%)*^*a*^	Chi square	*P* value	Bias	95% CI for bias	*P* value
25 (OH) D	random	2.20	1.38–3.02	5.25	< 0.001	92.3	39	< 0.001	9.02	6.97,11.07	0.003
1.25 (OH) D	Random	1.23	0.01- 2.44	1.398	0.048	86.3	7.32	0.007	-	-	-
PTH	Random	-0.75	-1.3—-0.18	2.57	0.010	82.4	11.38	0.003	-4.7	-72, 62	0.535

### Vitamin D and Parathyroid Hormone (PTH)

On the other side, the listed evidence saw a sizable decrease in Parathyroid hormone (PTH) (SMD = -0.75, *I*^*2*^ = 82.4, 95% Confidence interval = (-1.3) – (-0.18), *P* value =  < 0.010). As reported, heterogeneity in PTH was statistically significant.

### Adverse events

The adverse events were reported in two studies. Schroten et al. reported lymphoma and traumatic hip fracture in two patients separately. Moreover, upper airway infections were observed as the most fashionable adverse events in both groups by Bislev et al. [20, 24]. No data were reported in other studies.

**Fig. 5:**
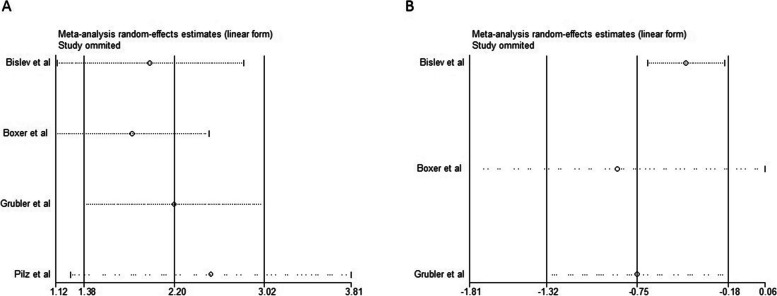
**A** Sensitivity analysis for SMD of 25(OH)D. **B** Sensitivity analysis for SMD of PTH

**Fig. 6:**
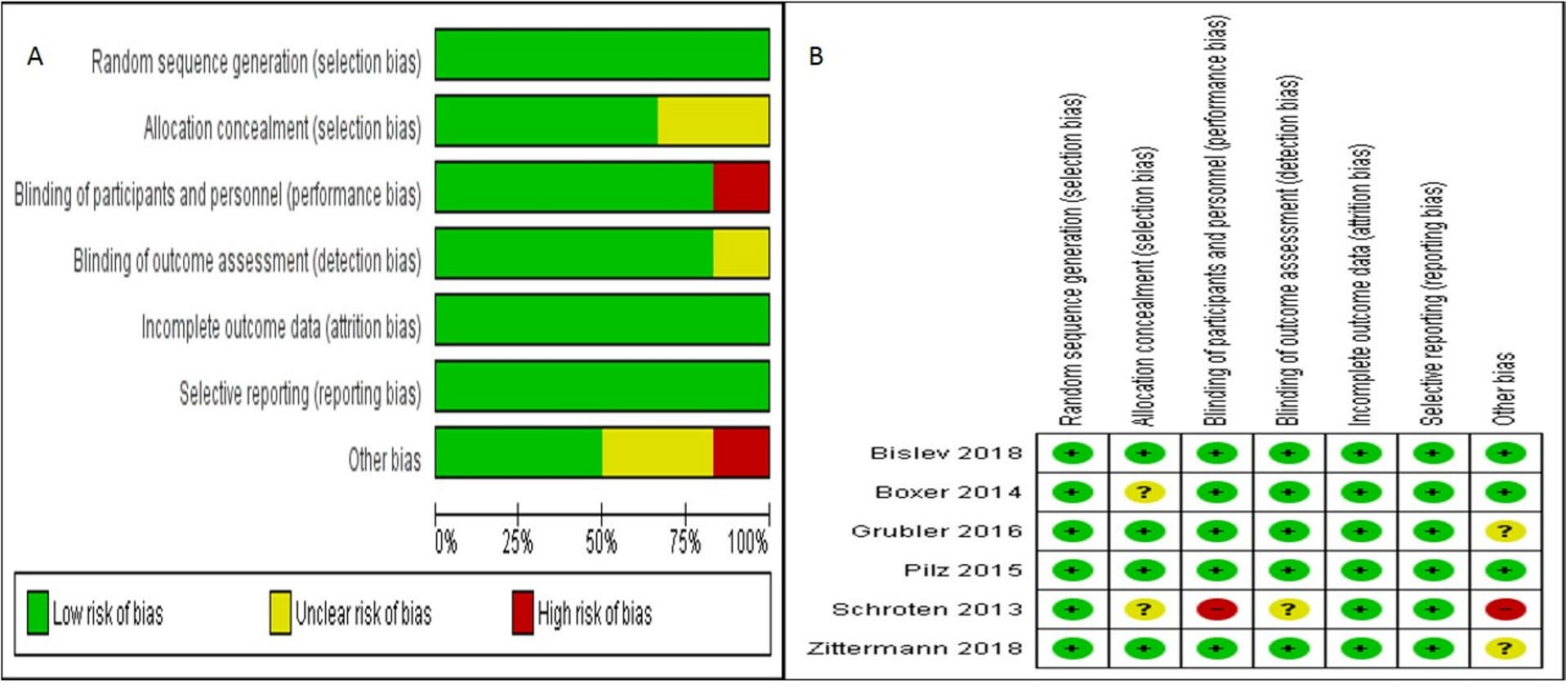
**A** Risk of bias graph. **B** Risk of bias summary

On the other hand, sensitivity analysis was performed for all three parameters, with a sensitivity analysis diagram to eliminate each of the studies in Fig. 5. The risk of bias is also shown in Fig. 6. In terms of 25(OH)D, the amount of publication bias was significant. According to the sensitivity analysis of the study of Pilz et al., it had the most impact on SMD results in 25(OH)D [23]. The study of Bislev et al. highly influenced the difference between the average standard PTH in the two groups [20].

## Discussion

Our analysis unveiled a notable decrease in parathyroid hormone (PTH) levels and significant improvements in 25(OH)D and 1,25(OH)_2_D levels in the intervention group compared to the control group, thereby substantiating the efficacy of vitamin D supplementation. 1,25(OH)_2_D has been shown to be a negative endogenous regulator of renin production in research on experimental animals [26]. However, despite extraordinarily high baseline renin levels, vitamin D did not reduce renin concentrations in Zittermann et al.'s research [25]. Similarly, in the other clinical trials, vitamin D supplementation did not reduce renin concentrations [21, 22, 27]. Vitamin D significantly reduced plasma renin in individuals with coronary artery disease and diabetes, according to two additional RCTs [28, 29]. The aforementioned study observed a significant rise in circulating 25(OH)D despite the administration of the active vitamin D hormone calcitriol (1,25(OH)_2_D3) at a dosage of 0.5 μg/day. However, there was no report of the circulating 1,25(OH)_2_D levels. Notably, Zittermann et al.'s study showed a significant increase in plasma renin in the participants with insufficient levels of 25(OH)D, owing to the impact of vitamin D [25]. This could be due to increased intestinal phosphate absorption, as evidenced by findings in laboratory animals fed a diet rich in phosphate content [30]. During the experimental study, dietary phosphate consumption increased 1,25(OH)_2_D levels, plasma renin concentrations, blood pressure, and left ventricular hypertrophy. Therefore, whether vitamin D can suppress human renin synthesis is still uncertain.

In previous studies that examined the impact of vitamin D supplementation on RAAS activity, divergent outcomes were discovered. A few of these trials demonstrated that consuming vitamin D supplements reduced plasma levels of aldosterone, renin, and blood pressure [22, 24, 31, 32].

However, McMullan et al. revealed that ingesting ergocalciferol (vitamin D2) did not significantly affect blood pressure or RAAS activity [15]. Moreover, no links between 25(OH)D and the RAAS were observed, and consequently, no effect on blood pressure level can be made. On the other hand, some studies contradict this explanation. These studies conducted experiments with vitamin D3 and did not observe any impact on cardiovascular measures [15, 20].

Furthermore, remarkably, it is believed that the elevation and sustenance of 25OHD levels are significantly more pronounced with vitamin D3 compared to vitamin D2, exhibiting a potency differential of at least threefold and quite possibly closer to tenfold [33].

The role of vitamin D in enhancing calcium absorption within the intestines is a known fact. However, most studies published thus far have not shown an increase in renal excretion due to vitamin D supplementation [23, 34, 35]. It was unexpected that Bislev et al.'s study demonstrated a 32% (95% CI: 16 to 49) rise in renal calcium excretion following vitamin D supplementation. This increase in urinary calcium may lead to an augmented risk of nephrocalcinosis and nephrolithiasis. Therefore, future investigations must evaluate this potential adverse effect in greater detail [36]. Maintaining calcium homeostasis mainly depends on renal calcium handling, as calcium levels can affect blood pressure regulation. Studies have established a tenuous connection between renal calcium excretion and most blood pressure measurements collected over a 24-h period [37, 38].

Various interventional studies have shown a clear correlation between parathyroid hormone (PTH) and aldosterone; however, most of these studies executed interventions that treated hypertension via antihypertensive medication or conducted adrenalectomy (in patients with primary hyperaldosteronism) [39–42]. Bislev et al. did not demonstrate a correlation between PTH and RAAS [20]. The present study investigated the effects of aldosterone blockade on individuals with primary hyperparathyroidism over a duration of 8 weeks, utilizing the selective mineralocorticoid receptor antagonist eplerenone [20]. The administration of eplerenone resulted in a decrease in blood pressure and a possible reduction in urinary calcium excretion. However, no significant effect was observed on the parathyroid hormone (PTH) levels. These findings challenge previous research suggesting a positive correlation between aldosterone and PTH levels. One explanation is that the link between the two may be due to hypertension, as lowering blood pressure can increase plasma calcium by reducing renal calcium and magnesium excretion, ultimately resulting in decreased PTH levels [20]. Future research should concentrate on delving deeper into these mechanisms.

In this particular discussion, the focus is on analyzing the impact of vitamin D on the RAAS system in patients or animals with specific medical conditions. In research using a mouse model of experimental colitis, Wei et al. investigated whether vitamin D inhibits the local colonic RAAS to reduce colonic mucosal inflammation [43]. This research showed that low vitamin D levels enhance colonic inflammation, at least in part because the colon's local RAAS is overactive. Butler et al. investigated if there is a link between vitamin D and RAAS in women with polycystic ovarian syndrome (PCOS) [44]. The lack of vitamin D can adversely impact the endocrine renin-angiotensin system (RAAS). Women with polycystic ovary syndrome (PCOS) often suffer from vitamin D deficiency, which can cause overactivation of RAAS in PCOS. This article revealed that PCOS subjects with insufficient or deficient vitamin D levels had an increased activation of RAAS due to higher plasma renin levels compared to non-PCOS control women. Krummel et al. performed a research study to examine if giving native vitamin D supplements could decrease albuminuria in chronic kidney disease (CKD) patients with stable conditions who were being treated with maximal renin-angiotensin system (RAAS) blockade [45]. The researchers discovered a significant but minor reduction (15%) in albuminuria after administering a large dose of vitamin D supplements. They did not see any changes in the systemic RAAS or blood pressure, which aligns with recent clinical studies. In 2020, another study assessed the impact of using RAAS inhibitors (RAASI) on patients with coronary artery disease (CAD) undergoing percutaneous coronary intervention (PCI) concerning their vitamin D levels [46]. The study discovered that in patients undergoing PCI, taking RAASI was linked to a reduced risk of major adverse cardiovascular events (MACE) only in individuals with lower vitamin D levels. These research findings highlight the significance of vitamin D in various diseases and signal healthcare practitioners to prioritize checking their patients' vitamin D levels.

## Conclusion

In conclusion, vitamin D supplementation was found to have a positive effect on serum levels of 25(OH)D and 1,25(OH)_2_D while decreasing parathyroid hormone (PTH) levels. While some studies reported decreasing effect of vitamin D supplements on RAAS activity, some reported no changes. Although vitamin D supplementation appears to be beneficial for improving vitamin D levels and reducing PTH levels, further studies are needed to assess the long-term effects and potential adverse events associated with vitamin D supplementation. Clinicians should carefully evaluate the risks and benefits of vitamin D supplementation for individual patients based on their specific medical history and health status.

### Limitations

In this study, although we could bring together the results of clinical trials and make a clear conclusion based on a pooled analysis of their results about the effect of vitamin D supplements on the serum level of 25(OH)D, 1,25(OH)_2_D, and PTH, the restricted number of randomized controlled trials (RCTs) meeting our inclusion criteria may affect the generalizability of our findings. Second, due to heterogeneity of the studies we could not make a pooled analysis on the effect of these supplements on RAAS activity and our conclusion limited to the systematic review of the reported studies. Third, the relatively short follow-up durations in some studies may not capture long-term effects and potential adverse events associated with vitamin D supplementation, particularly in the context of renal outcomes. Despite these limitations, our study offers valuable insights into the current understanding of the relationship between vitamin D supplementation and RAAS activity. Future research with larger sample sizes, standardized interventions, and longer follow-up periods is warranted to address these limitations and provide more conclusive evidence.

## Data Availability

Analysed data can be requested from the authors. Please write to the corresponding author if you are interested in such data.
